# Homesickness at Home: A Scoping Review of Solastalgia Experiences in Australia

**DOI:** 10.3390/ijerph20032541

**Published:** 2023-01-31

**Authors:** Matilde Breth-Petersen, Jasper Garay, Kaiwarr Clancy, Michelle Dickson, Candace Angelo

**Affiliations:** Sydney School of Public Health, Faculty of Medicine and Health, The University of Sydney, Camperdown, Sydney, NSW 2006, Australia

**Keywords:** solastalgia, Aboriginal and Torres Strait Islander, connection to Country, mental wellbeing, social and emotional wellbeing, place-based distress, psychoterratic, eco-psychology, environmental change, climate change

## Abstract

Solastalgia is a term used to describe the pain and distress experienced by those witnessing their home environments destroyed or changed in unwelcome ways. Solastalgia is expected to become more prominent as climate change worsens and transforms landscapes. This scoping review examines and maps the existing literature on solastalgia in Australia, particularly focusing on Aboriginal and Torres Strait Islander experiences. Four focus questions guided the review to explore how solastalgia is conceptualized, highlight risk and protective factors, and identify strategies for addressing solastalgia. Eighteen papers met the criteria for inclusion. Overall, our results show a minimal evidence base on solastalgia in Australia with an even greater gap in exploring solastalgia from Aboriginal and Torres Strait Islander perspectives. A strong connection to home environments was suggested as both a risk and protective factor for experiencing solastalgia. Aboriginal and Torres Strait Islander peoples are considered at risk due to intimate connections to home environments, and since the invasion, have experienced mental distress resulting from significant, damaging changes to landscapes and home environments. We recommend further exploration of lived experiences of solastalgia across a greater diversity of Australian contexts, particularly amongst Aboriginal and Torres Strait Islander peoples, including a focus on practical implications.

## 1. Introduction

The distress and pain felt by those who experience their home environments being destroyed or changed in ways that alter their sense of place is a concept known as solastalgia [1]. Albrecht coined the term in 2003 after recognising a decline in his own mental wellbeing while examining the impacts of mining in his home environment [1]. Solastalgia is a portmanteau of the words: solace (i.e., comfort), algos (i.e., pain or suffering) and nostalgia [2]. Thus, solastalgia refers to the pain and distress caused by the loss or inability to derive solace when there is the lived experience of physical desolation in a home environment [3]. External forces, like extreme weather events and mining activities, are common causes of landscape and seascape changes that can lead to feelings of psychological desolation about its transformation [3,4]. A lack of solace can erode our sense of place, belonging and identity through the disconnection and powerlessness felt in response to unwelcome environmental changes [3]. Albrecht describes nostalgia as intense distress and melancholia triggered by homesickness (when distant from home) [1]. As nostalgia and solastalgia both describe distress triggered by a compromised sense of place, solastalgia is often described as “homesickness at home” [3].

The incidence of solastalgia is expected to grow as weather and climatic events increase in frequency and severity with the worsening of climate change [5,6]. As global temperatures continue to rise, Australia is likely to experience more acute and chronic weather events, including flooding, bushfires, droughts and salinisation [7,8]. These events will alter landscapes and seascapes, which can be distressing to people as they connect with their home environments. This concept appears especially pertinent worldwide for First Nations peoples who have intrinsic cultural and spiritual connections to the land [9]. In Australia, Aboriginal and Torres Strait Islander peoples refer to this attachment as a connection to Country, whereby Country encapsulates a system that people both belong to and are related to [10,11], and an essential source of culture and identity [2,12]. Land and sea degradation potentially destroys cultural and spiritual connections and affects health and wellbeing in ways that most non-Indigenous people cannot comprehend [13,14].

Globally, the unique cultures, languages, worldviews, and social systems of First Nations peoples are often unrecognised or underacknowledged, within a history of land dispossession, human rights violations and other historical traumas that continue to intensify social, economic, and cultural disadvantages [15,16]. First Nations determinants of health and wellbeing vary from Western models of health, with the land, sea, and reliance on natural environments for cultural practices and livelihood recognised as vital for overall First Nations wellbeing [17,18]. As climate change and the global burden of mental wellbeing challenges are expected to intensify, these key determinants of health and wellbeing are becoming significantly disrupted, leading to increasing mental health impacts and inequalities for First Nations communities [19].

Links between poor social and emotional wellbeing and changes to natural environments have already been documented in First Nations populations worldwide [9,19]. Despite these realised and predicted impacts on mental wellbeing, there appears to be a scarcity of evidence to improve our understanding of solastalgia amongst Aboriginal and Torres Strait Islander peoples [20]. While the term ‘solastalgia’ is relatively new, it is critical to note that Aboriginal and Torres Strait Islander peoples, and other First Nations peoples around the world, have been experiencing this type of mental distress for many years since colonisation/invasion started to drive significant, damaging changes to the landscape and home environments. The gap in understanding First Nations’ experiences of solastalgia has been acknowledged by Galway and colleagues in the first review synthesising the existing solastalgia literature [6].

To date, there has been no review specifically mapping the Australian literature to describe the nature and extent of this negative emotional experience on Australian communities, where climate change is already rearing its ugly head, as seen by unprecedented bushfires, droughts, and flooding. This paper aims to bridge the research gap by mapping the literature on solastalgia in Australia. Our review aims to understand how solastalgia is conceptualised in Australian literature and identify various risks, protective factors, and strategies to address solastalgia in Australia. This paper has a particular focus on Aboriginal and Torres Strait Islander peoples’ experiences; as such, we aim to go beyond merely naming this group as at-risk, as much of the existing literature has done [6], and will attempt to explore the relationship in more depth. Four research questions guided this scoping review:

1. How is solastalgia defined and differentiated from other eco-psychological terms in Australian literature?
2. What are the risk factors and protective factors for experiencing solastalgia in Australia?
3. How is solastalgia experienced by Aboriginal and Torres Strait Islander peoples?
4. How can solastalgia be addressed in Australia?

## 2. Materials and Methods

Scoping reviews are commonly preferred when mapping literature in a novel field of research with limited available literature [21] Given that solastalgia is still recognised as a novel concept with the limited available literature, a scoping review approach was determined most appropriate for addressing this study’s broad research questions [21]. The methodological framework was guided by the Joanna Brigg Methods for Scoping Reviews [22], and the Preferred Reporting Items for Systematic reviews and Meta-Analyses extension for Scoping Reviews (PRISMA-ScR) Checklist [23].

The search strategy was developed through research team discussion (MBP, JG, CA, KC) and consultation with a university librarian experienced in health-specific research. A keyword search of four databases (Scopus, Environment Complete, PsycINFO, Informit) was conducted in late 2021 to address the research questions. The search was repeated (MBP, KC) to update results in September 2022. We used the following search terms: Solastalgi* OR ((climate OR eco* OR environment* OR sustainab*) w/4 (anxiety OR grief OR loss)) AND Indigenous OR Aborigin* OR “First Nations” OR “Torres Strait” AND Australia*. Backward citation chaining allowed the inclusion of additional papers not directly identified in the initial search. Notably, 80% of the authorship team (JG, KC, MD, CA) identify as First Nations people.

To determine whether each paper met the criteria for inclusion in the review (see criteria in Table 1), the titles and abstracts of search results were individually screened by two reviewers (MBP, KC). Any papers where inclusion was uncertain were subject to full-text screening, completed by three reviewers (JG, KC, MBP). Any disagreements on a paper’s eligibility were resolved in a group discussion with the rest of the research team. 

Reviewers (JG, KC, MBP) used a pre-determined Excel data extraction template (see Data Availability Statement below) to identify relevant data from the included papers to address the four focus questions. The data extracted from the included studies were: year, study context, population/participants, study type, aims and outcomes/key messages, and details relevant to answering each focus question.

Specific factors were considered when evaluating a paper’s relevancy to a focus question. Papers that defined solastalgia in detail, rather than just mentioning it, or defined other eco-psychological terms, were included to address focus question one. Papers that presented epidemiological data or emotive and reflective accounts from interviews or focus groups were included to address focus question two. To address focus question three, papers were scanned for phrases such as “connection to Country,” “social and emotional wellbeing”, “self-identity”, and “place attachment” to understand Aboriginal and Torres Strait Islander experiences of solastalgia. For focus question three, the explicit use of the term ‘solastalgia’ was not necessarily required to determine inclusion, given that the concept of place-based distress has shown relevance to Aboriginal and Torres Strait Islander wellbeing long before Albrecht coined ‘solastalgia.’ Importantly, only literature that described the distress associated with landscape or seascape changes while still at home was included to ensure specific relevance to solastalgia. Data on recommended strategies or interventions were extracted to address focus question four, mainly from discussion sections of included papers.

## 3. Results

**Figure 1:**
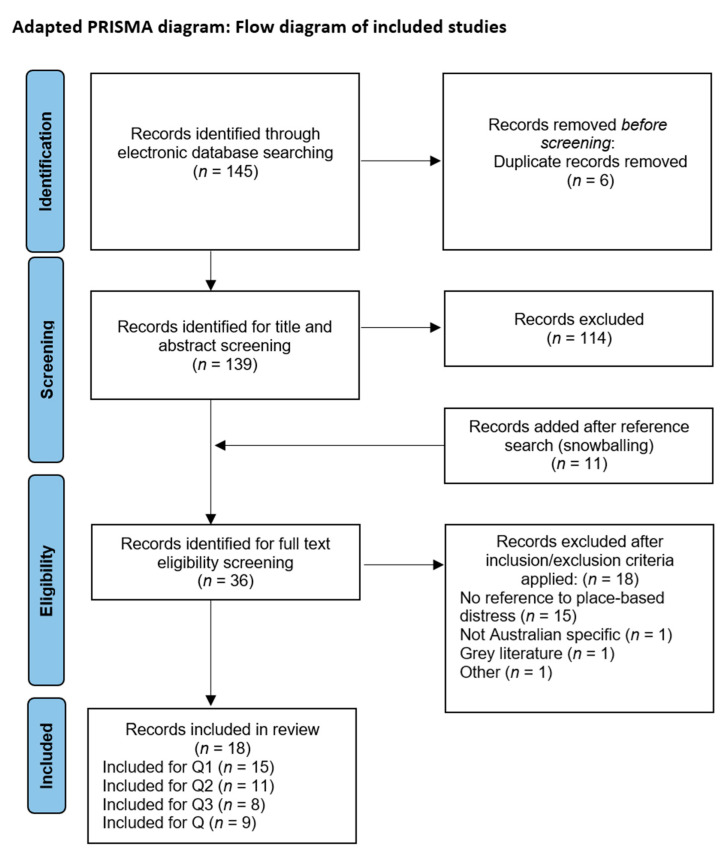
PRISMA diagram visually displaying search results.

The search returned 145 papers, 25 of which were eligible for full-text screening. Eleven additional papers were identified through backward citation chaining, creating 36 papers eligible for full-text screening. Of these, 18 papers met the criteria for inclusion in this scoping review. Search results are shown using an adapted PRISMA diagram (see Figure 1) [24,25].

### 3.1. General Characteristics of Included Papers

Of the 18 studies included in this review, 13 (72.2%) were primary studies, and five (27.8%) were perspective/viewpoint papers. All papers were specific to the Australian context. Seven (38.9%) were published in the past five years (2018–2022). Study types included ethnographic, observational, phenomenological, cross-sectional, and case studies (a total of 11 qualitative studies and one quantitative study). Four papers explicitly focused on Aboriginal and Torres Strait Islander communities, nine mentioned Aboriginal and Torres Strait Islander peoples, and five contained no mention of Aboriginal and Torres Strait Islander peoples. Included papers traversed multiple fields, including environmental health, public health, psychology, psychiatry, social sciences, and philosophy, demonstrating the multi-disciplinary nature of the concept of solastalgia. See Table 2 for a summary of the included paper characteristics (a more detailed summary of the 18 included papers is included in Appendix A, Table A1).

### 3.2. Addressing the Research Questions

#### 3.2.1. How Is Solastalgia Defined and Differentiated from Other Eco-Psychological Terms in Australian Literature?

In this scoping review, we aimed to gain an understanding of how solastalgia is defined and conceptualised in the included published literature. Of the 18 included papers, 15 papers explicitly defined solastalgia. Of the remaining three, one simply mentioned the term without a definition [26], and two did not mention the term at all [27,28] but rather described a profound sense of grief, despair and despondency amongst community members brought about by the declining condition of their home landscape.

##### Defining Solastalgia

Of the 15 papers that provided detailed definitions of solastalgia, seven were led or co-authored by Albrecht himself [1,2,3,29,30,31,32], one quoted Albrecht’s 2005 and 2007 definitions [33], five paraphrased Albrecht’s 2005 and 2007 definitions [13,34,35,36,37], and two extended and challenged them [38,39]. Wright disagrees with Albrecht’s assertion that solastalgia is linked to the present only [39]. Wright believes that focusing on the present is problematic because it ignores the ways in which the past and present interact. According to Wright, the solace we derive from a place comes from our memories and subsequent connection to the place, rather than its present condition only [39]. Askland and Bunn argued that place-based distress is triggered by several factors [38], such as social disruption and mistrust of industries and governments, and not just land degradation [38]. Moreover, Askland and Bunn insist that people’s primary concern is the ambiguity of their future [38].

All solastalgia definitions in the included papers were place-centric, with 12 papers (80%) using the term “place”, two (13.3%) using the term “home environment” [13,33], and one (6.7%) referring to the “home landscape” [37]. Only three (16.7%) of the papers defined place. Albrecht defined place as a biophysical space [3], and Askland and Bunn, as well as Wright, extended this to include society and memory, respectively [38,39].

Other words frequently used to define and conceptualise solastalgia include nostalgia (used in 10 papers, 66.7%), solace (9 papers, 60%) and homesickness (9 papers, 60%). As mentioned in the introduction, solastalgia is a portmanteau [2], so the frequent use of its constituent terms is expected. Similarly, solastalgia is often described as “homesickness at home” [2,3,29,30]. The phrase distils the essence of solastalgia into a pithy and memorable quote, which likely accounts for the extensive use of the term “homesickness” in the solastalgia-related evidence base. In the 18 included papers, common terms used to describe the emotional responses brought about by, or associated with, solastalgia were distress, despair, psychological desolation and melancholia brought about by environmental change or destruction. Other frequent emotion-related responses identified in the included papers were guilt, fear, helplessness and powerlessness at being unable to prevent unwelcome environmental transformation and being undermined by external forces, as well as concern, uncertainty and anxiety about the future of a transforming environment. Further, a feeling of dislocation and a declining sense of identity, belonging and familiarity was also evident across definitions in response to an attack on one’s attachment to place.

##### Other Eco-Psychological Terms

To better understand how solastalgia is conceptualised and applied in the Australian literature, we also explored other concepts mentioned in the included papers that relate to the emotional or mental health implications of deteriorating or changing environments. As described in four of the included papers, solastalgia is part of a broader ‘psychoterratic typology’, a classification system of both positive and negative earth-related emotions, based on the two-way connections between human and environmental wellbeing [3,29,30,32]. Solastalgia is described in these four papers as the opposite of topophilia, which is a term coined by Yi-Fu Tuan to describe a positive emotional experience of feeling joyful and at home in a healthy environment, or the affective bond (mental, emotional, and cognitive) between people and place [40]. When discussing topophilia, McManus and colleagues highlight that Aboriginal and Torres Strait Islander peoples and people living rurally typically have this more intense emotional connection to the landscape on which they live, and suggest that First Nations peoples are likely to use terms in their own language to describe these positive connections to the land [32]. 

Other eco-psychological terms used frequently in the literature include environmental distress, environmental anxiety, eco-anxiety, and ecological grief. Eco-anxiety was mentioned in two papers and was defined as “a sense of impending disaster or doom in relation to the natural world” [35]. Sartore and colleagues used the term “environmental distress”, however, this was not defined, and was used interchangeably with solastalgia. Environmental or ecological grief was also mentioned in one paper and was defined as a grief reaction stemming from the loss of landscapes, species and ecosystems caused by natural or anthropogenic events [3].

Only a few papers presented definitions of these related eco-psychological concepts or discussed the differences or similarities between them and solastalgia. A conceptual comparison of the aforementioned terms with solastalgia reveals a fundamental difference; that of place (defined here as the biophysical environment). Solastalgia is place-based distress that occurs when people experience changes to their home environment [29]. The other eco-psychological terms are more general and can affect those not experiencing environmental change first-hand.

#### 3.2.2. What Are the Risk Factors and Protective Factors for Experiencing Solastalgia in Australia?

##### Risk Factors

Of the 18 papers included in this review, 11 discussed numerous risks and protective factors for experiencing solastalgia. Humans with connections to specific geographic locations experiencing environmental degradation and alteration are suggested as particularly at risk. This risk factor featured in 36% of the total papers reviewed for this question [1,29,30,37]. Commonly identified risks were Aboriginal and Torres Strait Islander peoples with a deep connection to Country, colonial settler families possessing multi-generational ownership of agricultural and farming lands, rural and regional communities where extractive industries severely change the natural landscape, and the collective experience of industrialisation and urbanisation of communities [1,29,30,37].

Climate change was featured in four of the 11 articles relevant to this question. Particularly, impacts stemming from climate change that have tangible adverse outcomes on the home environment were identified as elevating the risk of solastalgia [1,29,30,37]. Experiencing drought, and witnessing negative changes to landscapes and waterways was found to heighten feelings of disconnection and displacement from what was previously a positive association with the home environment [37]. Both slow onset and extreme weather events have been identified as causational factors to various psychological and wellbeing effects [37]. Aboriginal and Torres Strait Islander peoples, rural communities, young people, and those from lower socio-economic backgrounds make up groups that are more susceptible to climate change-related experiences of solastalgia [1,26,37].

Adverse outcomes on community health and wellbeing, social cohesion, and individual factors were identified as risks resulting from, and contributing to, experiencing solastalgia [1,2,26,29,30,31,33,37]. Health issues alone featured as the largest risk factor; 45% of the papers assessed for this question identified health issues as a notable risk factor [1,2,29,31,37]. Population decline and loss of employment opportunities were found to impact community health, wellbeing, and cohesion [37]. For individuals, solastalgia has been described as having the potential to increase alcohol and illicit substance consumption and reliance on social supports, which have embedded reductions in positive connections to family and interpersonal relationships [1,37].

Various actions by local and state governments also presented as a risk factor contributing towards people’s experiences of solastalgia, appearing in 27% of the articles reviewed. Several issues stemming from governments’ handling of sustainability and planning, agricultural regulation, water management and mining leases were all seen as concerns contributing to experiences of solastalgia in communities [32,33,34].

##### Protective Factors

Protective factors for countering negative experiences of solastalgia were less commonly reported than were risk factors; however, a small number were identified within this review. In two of the included papers, Aboriginal and Torres Strait Islander concepts of connection to and caring for Country were suggested as an ethical model of understanding the importance of protecting environments that all Australian people should learn from [26,35]. Believing that humans have an innate duty to respect land, sea, airways, flora, and fauna through physical, mental, emotional, and spiritual caretaking, were approaches perceived to be of great relevance as protective factors for solastalgia. Similarly, ‘eco-cultural identity’, defined as the relationship between the human self and the ‘more-than-human’ world, was explained as a protective factor in one included paper due to the increased sense of responsibility and obligation to respect and care for flora, fauna, and environmental systems [35].

Civic engagement with planning against climate change through protest, education, and advocacy was identified as emerging yet meaningful mechanisms, serving as protective factors against solastalgia [26]. By doing so, individuals can collectively express shared concerns of negative feelings, receive information on the risks and what can be done about them, and potentially develop resiliency towards dealing with stressors that lead to experiencing solastalgia. Godden and colleagues concluded that health and wellbeing services should adopt climate change-appropriate intervention and assessment frameworks to deal with health and wellbeing challenges adequately [26]. They also identified that young people’s access to community groups and projects for combatting climate change may act as a support to mitigate climate change and consequently the onset of solastalgia [26].

#### 3.2.3. How Is Solastalgia Experienced by Aboriginal and Torres Strait Islander Peoples?

Eight of the 18 included papers related to Aboriginal and Torres Strait Islander experiences of solastalgia. The majority of the authors and co-authors reviewed in this focus question were found to be non-Indigenous to Australia. Co-authors that publicly identify as Indigenous contributed to just over a quarter (27%) of the literature that was reviewed for this focus question only [26,27].

Connection to Country appeared in all eight of the papers concerning this question and was described as a deeply profound and innate lived experience of Aboriginal and Torres Strait Islander peoples and their connection to the land, sea, animals, spirituality, kinship, and culture [1,13,26,27,28,29,36]. A further 85% of the papers relevant to this focus question described feelings of loss and sadness relating to the need to be on Country, but seeing their Country change before them [1,3,13,27,28,36]. The use of maladaptive coping mechanisms such as alcoholism and gambling were found to be used as a response to solastalgia-related distress from the changing land and Country [1,27], which, for Aboriginal and Torres Strait Islander peoples, directly results from the ongoing effects of colonisation [1]. 

In 57% of the papers reviewed for this question, the destruction of Country was named as leading to a loss of culture and lore [13,27,28,36]. James Ingram Jr tells of significant cultural practice in the Wiradjuri nation in New South Wales, Australia, where Wiradjuri boys learnt rope-making through their mothers, using a wetlands plant called Cumbungi [28]. The deterioration of wetlands has limited the availability of Cumbungi, preventing this cultural learning; the loss of such practices is devastating to Wiradjuri culture, having a detrimental impact on the community and individual wellbeing [28]. Women Elders from the Torres Strait Islands have also noted the devastation caused by rising sea levels to their harvesting of certain shells and foods integral to their culture [13]. They explicitly communicate the sadness and confusion that this causes them, which the authors relate to experiences of solastalgia [13]. The topic of solastalgia-related suicide, in relation to Aboriginal and Torres Strait Islander peoples, appeared once in Albrecht’s original paper [1]. Despite detailed discussion about how Aboriginal and Torres Strait Islander disadvantage and dispossession may be connected to suicide in Aboriginal and Torres Strait Islander communities [1], Albrecht mentioned no explicit evidence linking solastalgia or place-based distress as a contributing factor.

#### 3.2.4. How Can Solastalgia Be Addressed in Australia?

Of the 18 papers explored in this review, nine had made suggestions for addressing or mitigating the impacts of solastalgia on the mental health and wellbeing of the community [1,13,26,27,30,31,32,35,37]. These recommendations all similarly suggest that any potential intervention needs to be place-sensitive, resilience-building, and embrace traditional knowledge from Aboriginal and Torres Strait Islander peoples.

Three of the reviewed papers explored strategies related to resilience building [1,27,35]. Eco-cultural identity pertains to human connection to the world outside of humanity itself, for example, plants, seas, and soil [35]. As mentioned earlier, Boyd and Parr suggest that a positive eco-cultural identity may help address or reduce the distress associated with solastalgia [35]. They further suggest that this will build resilience by developing a stronger sense of agency regarding the changing climate [35]. Sartore and colleagues propose the development of community resources such as mental health support, men’s groups, and practical support for farmers as ways to build resilience in rural communities [37].

Place-based interventions were a common feature of the recommendations for addressing solastalgia, making up for 77.7% of the findings relating to this question [1,26,30,31,32,35,37]. A caring for Country (Indigenous land management) approach was featured as a method to reduce solastalgia and poor mental health in the community [26,27]. Rigby and colleagues propose that improving access to Country through the state forests may also increase wellbeing through the Caring for Country method [27]. Ellis & Albrecht did not mention addressing solastalgia specifically, however, they mentioned that mental health interventions for rural populations should be ‘place-based’ and could build upon lessons learned in Indigenous contexts that recognise the importance of place for health and wellbeing [30]. Further, Ellis & Albrecht also indicated that Natural Resource Management interventions should be explored with farmers to test claims that such initiatives improve the health of the land and farmers [30].

Indigenous cultural knowledge and connection to place/Country featured in 44.4% of the papers reviewed for this focus question. Godden and colleagues argue for culturally safe and appropriate strategies that support and increase resilience to the psychological impacts of climate change [26]. Further, interventions must be grounded in the local Country/place, landscapes and culture, and be responsive to other local contexts [26]. Rigby and colleagues recommend that Aboriginal and Torres Strait Islander peoples should be resourced and supported to develop land care programs that strengthen the connection to Country and protect against solastalgia [27]. Further, Rigby and colleagues suggest that Indigenous-led programs and events, such as the Koori Knockout and “pitstop” program for men, will be effective in improving mental wellbeing during times of drought [27]. In their Torres Strait Island study, McNamara and Westoby suggest that Torres Strait Islander communities that have adapted to the changes in the landscape have been able to maintain their sense of identity and place [13]. McNamara and Westoby also discuss the gendered perspectives of climate change as communicated by Torres Strait Islander ‘Aunties’ (older women) in their study and echo the United Nations Development Program’s recommendation that women should be included as active participants and decision-makers to ensure the effective development of climate-related adaptation strategies [13]. 

## 4. Discussion

Although the concept of solastalgia was coined by Albrecht almost two decades ago [1], it has only recently been acknowledged as an important impact of climate change on human health and wellbeing. Consequently, there has been a lack of comprehensive research to further develop our understanding of solastalgia [6]. This review aimed to identify and synthesise the literature pertaining to experiences of solastalgia in Australia, to gain a more in-depth understanding, and to highlight critical knowledge gaps.

By identifying ways in which the included literature conceptualises the term, we recognise that Albrecht’s definition of solastalgia appears most cited or paraphrased; however, it has been disputed by some who oppose its temporality and place definition. Risk factors for experiencing solastalgia were identified as being acute and chronic extreme weather events relating to climate change (for example, drought), collective experiences of industrialisation and urbanisation, and government actions relating to sustainability, planning and regulatory/management decisions. Narrative evidence from the included literature revealed that cultural cohorts (for example, Aboriginal and Torres Strait Islander peoples) and professional cohorts (for example, farmers) connected to the land are most at risk of solastalgia, although no quantitative evidence exists to strengthen this assertion. Importantly, connection to and caring for home landscapes and seascapes was suggested as both a risk and a protective factor for experiencing this form of place-based distress, with eco-cultural identity and engaging in advocacy movements identified as additional protective factors. While several strategies to address or mitigate solastalgia have been suggested in the included literature that are place-sensitive, resilience-building, and harnessing traditional knowledge, none have been implemented or assessed for effectiveness.

Within the context of Aboriginal and Torres Strait Islander experiences of solastalgia, the destruction of Country was identified as leading to the loss of culture and lore, with certain traditional practices no longer possible. These cultural impacts contribute to detrimental impacts on mental health, belonging, cultural identity and social and emotional wellbeing. Given the limited data available specific to Aboriginal and Torres Strait Islander experiences of solastalgia, it was not possible to obtain a more in-depth understanding of how this psychoterratic concept is applied and conceptualised among this group. International solastalgia literature is likewise limited when discussing First Nations experiences, however, there are some similarities to our findings [19]. For example, Indigenous peoples of Kiribati and other island countries in the Pacific Region have been reported to be experiencing distress due to the imminent threat of land loss from alarming levels of rising sea levels, similar to the events seen in the Torres Strait Islands in far Northern Australia [13,41,42,43,44]. Another example is Indigenous farmers in rural KwaZulu-Natal regions of South Africa, where environmental changes (soil infertility, erosion and drought) have limited food production [45]. These experiences are driving feelings of solastalgia, amplified by intrinsic place-based attachment and strong kinship bonds comparative to Aboriginal and Torre Strait Islander peoples [45]. Whilst climate change is recognised as driving these landscape changes, soil erosion is also linked to a major dam developed under the occupation of white colonial rule in South Africa [45]. This link supports our findings that colonisation and climate change are both significant factors concerning First Nations peoples’ experiences of solastalgia [28,45].

### 4.1. Implications

The findings of this scoping review uniquely build on the findings of the work by Galway and colleagues, by providing an Australian-specific picture of the solastalgia evidence base [6]. The results suggest several theoretical and practical implications. The definition of solastalgia itself appears to have strayed little from how Albrecht conceptualised it almost two decades ago [1]; however, our findings show that its temporality and definition of place have been contested. Our definition-related findings summarise the emotions associated with solastalgia, as described in Australian literature, its relationship to a sense of self, belonging and familiarity, and highlights the importance of ‘place’ as a conceptual comparison to other eco-psychological terms [3,29], all consistent with the findings of Galway and colleagues [6]. The absence of research and political action could partly be attributed to our present understanding of solastalgia as an experience or feeling, rather than a condition that warrants intervention. Askland and Bunn argue that positioning solastalgia as a diagnosable mental health condition, rather than an experience or feeling, may encourage institutional action, and empower sufferers [38]. Further, lobbying for institutional intervention against it could thrust it into the political sphere and initiate a mitigatory response [38]. On the other hand, viewing solastalgia as a mental health condition could be contested as medicalising a social condition. A comparative perspective is seen with ‘Ulysses Syndrome’, a depathologised term often used to describe experiences of war refugees for whom suffering is an understandable and direct result of extreme conditions, not a psychological disorder [46]. In the case of Aboriginal and Torres Strait Islander peoples, social and emotional wellbeing is often preferred to Western models of diagnosable mental illnesses [47]. Hence, a perspective of solastalgia as a social issue or condition could be viewed as more inclusive and lead to holistic social interventions (e.g., social support, training, employment) rather than medical or psychological interventions [46].

Our findings also summarise the specific factors that may lead individuals or communities in Australia to be particularly at risk of, or protected against, experiencing solastalgia; that data may, in turn, help address the issue. Many solastalgia studies focus on climate change as a primary risk factor, which was also seen in this review (i.e., drought); however, several findings describe risks unrelated to extreme weather. Other risks identified were related to colonisation, extractive industries’ actions and governmental decisions concerning destructive activities (for example, land clearing) [32,33,34]. Our findings also highlight an apparent two-way causal relationship between solastalgia and social cohesion, interpersonal relationships and negative health and wellbeing [1,37].

Another main finding was that connection to (and caring for) Country, is recognised as both a risk and a protective factor of experiencing solastalgia, within an Aboriginal and Torres Strait Islander context [18,35,37]. Therefore, it is essential to recognise that having intrinsic connections to a home environment can lead to experiencing solastalgia, but also equally important to harness this sense of responsibility and respect for the natural environment when addressing these emotional responses. Importantly, also, the finding that addressing solastalgia through fostering deeper notions of eco-identity and caring for Country could, in turn, help mitigate climate change, given that people may be more likely to engage in advocacy efforts and take action to protect their natural environment [18,26,35,37].

### 4.2. Strengths

To the best of our knowledge, this is the first scoping review specifically on solastalgia in Australia, and only the second solastalgia-related scoping review to have been conducted to date. A key strength of this review is the specific focus on Aboriginal and Torres Strait Islander experiences and perspectives. Much of the existing solastalgia literature appears to mention First Nations perspectives as an afterthought, or has First Nations peoples named merely as one of the ‘at-risk’ groups, without undertaking further in-depth exploration. Our paper prioritised this as a focal point throughout, whilst also capturing non-Indigenous studies and groups at risk. Given this focal point, we were able to refine our search strategy to capture in our search four Aboriginal and Torres Strait Islander-specific papers, two of which described the intense sense of grief and despondency concerning environmental degradation underpinning the concept of solastalgia without explicitly mentioning the term [27,28]. The review by Galway and colleagues highlights strongly an absence of solastalgia-related literature authored by First Nations scholars [6]. This current review has been co-authored by a majority First Nations team, allowing us to apply a First Nations lens that helps communicate a genuine and authentic understanding of Aboriginal and Torres Strait Islander experiences of solastalgia [6].

Other strengths worth mentioning pertain to our methodology, including obtaining advice regarding the search strategy from an experienced health sciences librarian. Further, two independent reviewers assessed the papers for inclusion, and two independent reviewers extracted data that was then verified by a third reviewer, which helped to enhance the robustness of our findings by minimising bias.

### 4.3. Limitations

There are several limitations to note. One is that no formal critical appraisal of included studies was conducted. However, this is not required based on the guidelines of scoping reviews, particularly given that such reviews do not aim to assess the quality of the papers included. Secondly, we decided to cap the criteria for inclusion in the year 2003, when Albrecht first coined the term solastalgia [1]. However, we acknowledge now that this contradicts part of the aims of this review, which was to understand Aboriginal and Torres Strait Islander experiences of the concept of place-based distress. Earlier, we noted that such distress has been felt for a lot longer than the term solastalgia has been around, occurring for hundreds of years because of invasion and continuing impacts of colonialism, so including literature prior to 2003 may have helped to shed further light on these impacts. Further, we strictly included articles describing distress regarding environmental changes whilst still connected to home to ensure we reflected the concept of solastalgia as accurately as possible. However, we recognise that Albrecht himself has briefly postulated that this emotional experience may also be possible in people identifying Earth more broadly as their home rather than simply the home or land on which one resides [1]. This notion contends that the distress felt whilst witnessing environmental destruction in any place on Earth (for example, via media broadcasts of land clearing or bushfires) could also be described as solastalgia. Viewing solastalgia through this lens would have allowed us to capture a broader range of solastalgia experiences, such as amongst those displaced from their home environments and unable to reside on Country or their home environment (and thus, unable to care for Country) [48]. Given the lack of clarity around this conceptualisation, we chose to use the working definition of distress whilst still at home. Lastly, we did not include any grey literature, which means there may be community, practice, or policy-focused literature, or fictional, media, or art relating to solastalgia not captured in this review that could have provided more holistic socio-cultural and practical understandings of the concept. These various limitations should be acknowledged when considering the implications of the review’s findings.

### 4.4. Recommendations for Future Research Directions

In terms of future research, it would be useful to extend the current findings by examining a more comprehensive range of population groups and geographical locations across Australia. The vast majority of papers focused on rural or regional New South Wales, with the remainder investigating solastalgia in communities of Western Australia, the Northern Territory and the Torres Strait Islands, leaving the remaining Australian states and territories unexplored to date. Based on the literature included in this review, solastalgia in metropolitan environments is hardly considered despite its relevancy to urbanisation, and thus little is known about the effect of urbanisation on place attachment in relation to solastalgia. Diverse methodologies, particularly involving mixed methods and epidemiological data, are also needed to strengthen narrative evidence and understand the breadth and extent of solastalgia in Australia, which may help to get solastalgia on the political agenda. Further, as mentioned earlier, there is a gap in understanding the practical implications of solastalgia and how we can best intervene, raising the need for research to explore strategies or interventions to address solastalgia.

There is a need for a better understanding of how solastalgia is perceived, conceptualised, and applied in Aboriginal and Torres Strait Islander communities, which must be acknowledged in the context of historical traumas and the holistic impacts (i.e., spiritual, cultural, social and emotional wellbeing). Importantly, given the power and complexity of language [20], there is a further need for research into whether the term is indeed accurate or inappropriate for describing Aboriginal and Torres Strait Islander experiences. Albrecht has suggested solastalgia as a more inclusive and culturally appropriate eco-psychological term for Aboriginal and Torres Strait Islander peoples, than terms with the prefix “eco” (for example, eco-anxiety or ecological grief) that force Western science onto Indigenous belief systems and wellbeing, identified by Albrecht as a form of neo-colonialism [3]. One First Nations scholar, consulted for the 2018 review by Galway and colleagues, held the belief that there are better concepts to use as “solastalgia is a colonised word, and using the term solastalgia (to describe Indigenous experiences) feels like trying to knock a square peg into a round hole” [6]. An example of this is the community-driven study by Rigby and colleagues, included in this study, which described the place-based distress felt by Aboriginal and Torres Strait Islander peoples without using the term solastalgia [27]. Therefore, we echo Galway and colleagues’ recommendations that solastalgia-related research should be conducted using decolonising research methodologies and led or conducted in collaboration with Aboriginal and Torres Strait Islander communities to better understand whether the community feel the term is appropriate and beneficial for describing the lived negative experiences of environmental change [6]. Similarly, we recommend that future research to address the gap in solastalgia interventions should also prioritise a community-driven approach to ensure the appropriateness of such interventions [49]. Encouragingly, two of the Aboriginal and Torres Strait Islander-specific papers included in this review were co-authored by researchers who have publicly identified as Aboriginal [26,27]; we look forward to further Aboriginal and Torres Strait Islander-led research on this topic.

Several unanswered questions resulting from this review are worthy of future research. One is whether conceptualising solastalgia as a diagnosable mental health condition may help spread awareness of its negative emotional impacts and, notably, support mental health workers in addressing its symptoms. A second question remains about whether raising public and political awareness of this new concept and the threat it brings, may open peoples’ eyes more to acting against climate change. Or would this awareness raising simply add unnecessarily to the growing list of psychoterratic and eco-psychological terms, leading people to disconnect or lack interest? The final unanswered question resulting from this review should focus on understanding the potential widespread and long-term impacts solastalgia could have on Australian communities.

## 5. Conclusions

In summary, our scoping review of 18 Australian studies has drawn attention to, and mapped, the existing knowledge and understanding of solastalgia in the Australian context. Our review has importantly highlighted that, despite recent novel literature on the topic, there is still much to be learned about solastalgia, particularly from the perspectives of Aboriginal and Torres Strait Islander experiences and worldviews. As climate change worsens, solastalgia is expected to become increasingly relevant to all Australians. Further research is needed to heighten the awareness of solastalgia and other negative impacts of climate change and to further explore practical implications through community-driven mental health interventions and mitigatory responses.

## Figures and Tables

**Table 1:** Inclusion and Exclusion criteria.

Inclusion Criteria	Exclusion Criteria
Peer-reviewed	Published before 2003
Published in English	Not specific to the Australian context
Published between 2003 ^1^–2022	Grey literature Secondary literature (i.e., reviews)
Specific to the Australian context	
Addresses 1/more focus questions	

^1^ The year 2003 was chosen as the starting year for the inclusion criteria given that this was the year ‘solastalgia’ was first coined (Albrecht, 2005) [1].

**Table 2:** General Characteristics of Included Papers.

Characteristic	No. (%)
**Year of publication**	
<2010	5 (27.8%)
2010–2017	6 (33.3%)
2018+	7 (38.9%)
**Region in Australia**	
Broad/multiple regions	3 (16.7%)
New South Wales	11 (61.1%)
Western Australia	2 (11.1%)
Northern Territory	1 (5.6%)
Torres Strait Islands	1 (5.6%)
**Study type**	
Qualitative (Case study)	7 (33.3%)
Qualitative (Phenomenological)	2 (11.1%)
Qualitative (Observational)	2 (11.1%)
Qualitative (Ethnographic)	1 (5.6%)
Quantitative (Cross-sectional)	1 (5.6%)
Theoretical/conceptual (Perspectives)	5 (27.8%)

## Data Availability

The data presented in this study are openly available in Open Science Framework (OSF) at https://osf.io/qxe3n/?view_only=10e4fdff38984879bf4e41eb9fa77c01, last accessed on 28 December 2022.

## Appendix A

**Table A1:** Table summarising papers included in this review (author, title, date, aims/objectives).

Author	Title	Date	Aims/Objectives
Albrecht G., Sartore G.-M., Connor L., Higginbotham N., Freeman S., Kelly B., Stain H., Tonna A., Pollard G. [29]	Solastalgia: The distress caused by environmental change	2007	To explore and contextualise solastalgia
Albrecht, G. [3]	Negating Solastalgia: An Emotional Revolution from the Anthropocene to the Symbiocene	2020	To demonstrate that a shift in epochs, from one characterised by negative earth emotions to another characterised by positive ones, will alleviate feelings of solastalgia
Albrecht, G. [1]	Solastalgia: a new concept in health and identity	2005	To focus on two contexts where collaborative research teams have found solastalgia to be evident: the experiences of persistent drought in rural NSW and the impact of large-scale open-cut coal mining on individuals in the Upper Hunter Valley of NSW.
Askland H.H., Bunn M. [38]	Lived experiences of environmental change: Solastalgia, power and place	2018	To critically engage with the topic of solastalgia including consideration of power and dispossession.
Boon P.I. [34]	The environmental history of Australian rivers: A neglected field of opportunity?	2019	To explain how and why environmental change occurs from a cultural, rather than an ecological, perspective
Boyd C.P., Parr H. [35]	Climate change and rural mental health: a social geographic perspective	2020	To demonstrate the ways in which rural mental health can be enhanced by social geographic perspectives, in the context of climate change-induced pathologies
Connor, L., Albrecht, G., Higginbotham, N., Freeman, S. & Smith, W. [2]	Environmental Change and Human Health in Upper Hunter Communities in New South Wales, Australia	2004	To explore the proposition that the psychological wellbeing of individuals and their sense of place identity have been challenged by perceived degradation and transformation of the local landscape
Conroy C., Knight A.R., Wassens S., Allan C. [28]	Consequences of changed water management for Aboriginal Australians in the Murrumbidgee catchment, NSW	2019	To explore the understandings and perceptions of Aboriginal peoples in relation to the changes to their Country, water and the allocation of water for cultural and other uses.
Dixon J., Isaacs B. [33]	There’s certainly a lot of hurting out there: Navigating the trolley of progress down the supermarket aisle	2013	To understand how the supermarket institution contributes to the dynamics of a regional agricultural community undergoing profound structural change and consider the presence and responses to solastalgia.
Ellis N.R., Albrecht G.A. [30]	Climate change threats to family farmers’ sense of place and mental wellbeing: A case study from the Western Australian Wheatbelt	2017	To examine climate change as a mental health stressor amongst Australian family farmers, and to extend the application of place-related understandings of mental health and wellbeing to a non-indigenous and relatively affluent population
Godden N.J., Farrant B.M., Yallup Farrant J., Heyink E., Carot Collins E., Burgemeister B., Tabeshfar M., Barrow J., West M., Kieft J., Rothwell M., Leviston Z., Bailey S., Blaise M., Cooper T. [26]	Climate change, activism, and supporting the mental health of children and young people: Perspectives from Western Australia	2021	To examine the intersection of mental health, climate change, children and young people and climate activism in Western Australia as a resilience strategy
Higginbotham N., Connor L., Albrecht G., Freeman S., Agho K. [31]	Validation of an environmental distress scale	2006	To validate the Environmental Distress Scale.
McManus P., Albrecht G., Graham R. [32]	Psychoterratic geographies of the Upper Hunter region, Australia	2014	To explore the theoretical psychoterratic Concepts, topophilia and solastalgia, and their relationship to the field of impact assessment
McNamara K.E., Westoby R. [13]	Solastalgia and the gendered nature of climate change: An example from Erub Island, Torres Strait	2011	To explore the gendered nature of climate change and provide perspectives on how environmental impacts are experienced and responded to
Pannell S. [36]	Framing the Loss of Solace: Issues and Challenges in Researching Indigenous Compensation Claims	2018	To discuss how the issues of gender, resource development, environmental transformation, the Stolen Generation, and the history of Indigenous-European relations in remote and rural Australia impact upon investigations into the loss or diminution of traditional attachments to land… [and] explore some of the ‘conceptual challenges’ and methodological issues confronting anthropological investigations into compensation and the impact of impairment and extinguishment of Indigenous native title rights and interests
Rigby, C., Rosen, A., Berry, H. & Hart, C. [27]	If the land’s sick, we’re sick	2011	To report Aboriginal communities’ views of how prolonged drought in rural NSW has affected their social and emotional wellbeing, and possible adaptive strategies.
Sartore, G-M., Kelly, B., Stain, H., Albrecht, G., Higginbotham, N. [37]	Control, uncertainty, and expectations for the future: a qualitative study of the impact of drought on a rural Australian community	2008	To explore the stressors and difficulties that farming communities experience during prolonged drought
Wright, K. [39]	Pining for the Present: Ecological Remembrance and Healing in the Armidale State Forest	2012	To provide an alternative temporal framework that makes space for emotional attachments to an exotic timber plantation
